# Metacognition and action: a new pathway to understanding social and cognitive aspects of expertise in sport

**DOI:** 10.3389/fpsyg.2014.01155

**Published:** 2014-10-16

**Authors:** Tadhg E. MacIntyre, Eric R. Igou, Mark J. Campbell, Aidan P. Moran, James Matthews

**Affiliations:** ^1^Department of Physical Education and Sport Sciences, University of Limerick, LimerickIreland; ^2^Department of Psychology, University of Limerick, LimerickIreland; ^3^School of Psychology, University College Dublin, DublinIreland; ^4^School of Public Health, Physiotherapy and Population Science, University College Dublin, DublinIreland

**Keywords:** metacognition, expertise, cognition, motor cognition, social cognition, cognitive neuroscience, sport, sport psychology

## Abstract

For over a century, psychologists have investigated the mental processes of expert performers – people who display exceptional knowledge and/or skills in specific fields of human achievement. Since the 1960s, expertise researchers have made considerable progress in understanding the cognitive and neural mechanisms that underlie such exceptional performance. Whereas the first modern studies of expertise were conducted in relatively formal knowledge domains such as chess, more recent investigations have explored elite performance in dynamic perceptual-motor activities such as sport. Unfortunately, although these studies have led to the identification of certain domain-free generalizations about expert-novice differences, they shed little light on an important issue: namely, experts’ metacognitive activities or their insights into, and regulation of, their *own* mental processes. In an effort to rectify this oversight, the present paper argues that metacognitive processes and inferences play an important if neglected role in expertise. In particular, we suggest that metacognition (including such processes as “meta-attention,” “meta-imagery” and “meta-memory,” as well as social aspects of this construct) provides a window on the genesis of expert performance. Following a critique of the standard empirical approach to expertise, we explore some research on “metacognition” and “metacognitive inference” among experts in sport. After that, we provide a brief evaluation of the relationship between psychological skills training and metacognition and comment on the measurement of metacognitive processes. Finally, we summarize our conclusions and outline some potentially new directions for research on metacognition in action.

## INTRODUCTION

Expertise is characterized by superior reproducible performances and “refers to the characteristics, skills, and knowledge that distinguish experts from novices and less experienced people” (Ericsson, 2006a, p. 3). Quintessentially, sport provides many such instances. For example, when Lewis (Lewis and Marx, 1996) became the first track and field athlete to win four consecutive Olympic titles, he accomplished this feat with a long jump of 8 m 50 cm, winning by a margin of 21 cm. Sport is a domain that provides benchmarks to distinguish experts from novices, through performance outcomes (e.g., podium placing), player statistics (e.g., batting average in baseball) or competition level (e.g., Olympic vs. Collegiate). Given such criteria, it is not surprising that the question of how one becomes an expert within the sport domain has been of increasing scientific (Ericsson, 2014; Hambrick et al., 2014a) and popular interest (Ross, 2006; Gladwell, 2008) in the two decades since Ericsson’s seminal paper on deliberate practice. “Deliberate practice presents performers with tasks that are initially outside their current realm of reliable performance, yet can be mastered within hours of practice by concentrating on critical aspects and by gradually refining performance through repetitions after feedback” (Ericsson, 2006b, p. 692). Although subject of much debate (e.g., Baker and Young, 2014; Detterman, 2014), the theory of deliberate practice and the development of expertise both warrant further analyses. This paper adds to the expertise debate by presenting a novel argument contending that metacognitive processes are central to expertise in the sport context. Furthermore, we suggest that some of the aforementioned controversies in the research literature, that stem from conflict between those focused largely on exploring the role of automaticity in skilled performance, and researchers focused on understanding the representation of experts knowledge, may have led to the explanatory role of metacognition being overlooked.

In this review we propose that our understanding of aspects of both social and cognitive dimensions of sporting expertise can be adequately explained from a metacognitive perspective. The potential of metacognitive inferences and domain-general skills including psychological skills training (PST) are posited as integral to the genesis of expert performance. Subsequently, the contribution of both mental imagery (e.g., mental practice) and attentional strategies (e.g., routines) to our understanding of expertise and metacognition will be discussed. Finally, new directions for future research that emanate from our metacognitive perspective on sporting expertise will be outlined. Firstly, however, in the rationale for our approach we will attempt to answer the following questions: are there limits to the current expertise approach? Why is sport an appropriate field of study? And finally, what is metacognition?

## ARE THERE LIMITS TO THE EXPERTISE APPROACH?

Historically, the rich tapestry of research on expert performance has been interwoven with a common thread-the study of grandmasters in chess (Williams and Ericsson, 2005). Investigations into expertise in chess, a competitive sporting activity that was rule bound, amenable to measurement through objective ratings (e.g., ELO rankings), with a range of possible contextual requirements (e.g., blindfold chess; Campitelli and Gobet, 2005) led to a proliferation of literature on the topic (de Groot, 1965; Chase and Simon, 1973; Holding, 1985, 1992). Chess is a challenge of perceptual-cognitive skill and thus provides a fitting laboratory for testing constructs such as pattern recognition, visual imagery, and memory (Bilalic et al., 2010). Sport in the more traditional sense emphasizes motor skill execution under stressful conditions typically in a dynamic environment (Baker and Young, 2014). One legacy of the chess expertise literature was that this perceptual-cognitive lens was subsequently applied in the sport domain (MacIntyre et al., 2013). Two interlinked events led researchers to become enthusiastic in their study of visual cognition and sport (Williams and Ford, 2007). Firstly, the emergence of the expert performance approach (Ericsson and Smith, 1991) and later, the theory of deliberate practice (Ericsson et al., 1993), both provided an impetus for investigations into perceptual-cognitive skills, such as anticipation and decision-making in sport. The tenet that sport could be a dynamic natural laboratory was well made (Moran, 1996; Williams and Ericsson, 2005) and the development of innovative methodologies occurred in parallel (e.g., eye-tracking as a measure of attention). A burgeoning literature developed and sport as a domain of study gained popularity as a result (Moran, 2009; MacIntyre et al., 2013).

However, there were limits to this approach, particularly in the focus on visual-cognitive expertise, which arguably was to the detriment of our understanding of the underlying psychological processes. Take, for example, the quiet eye phenomenon which has recently gained prominence in sport science research (Vine et al., 2014). Increasingly, this is becoming a topic of interest within both cognitive psychology (Klostermann et al., 2013) and neuroscience (Vine et al., 2011). The quiet eye is defined as “a final fixation or tracking gaze that is located on a specific location or object in the visuomotor workspace within 3∘ of visual angle (or less) for a minimum of 100 ms” (Vickers, 2007, p. 11) prior to the onset of a critical movement. According to Vickers, quiet eye offset occurs when the gaze deviates off a specific location for more than 100 ms (Vickers, 2007, p. 11). Despite the success in establishing a quiet eye phenomenon “there has been a lack of explicit tests of the processes through which quiet eye training interventions exert their positive effect” (Vine et al., 2014, p. S237). To date, little knowledge of the psychological basis of the quiet eye phenomenon has emerged (Moran, 2012a). Similarly, while a wealth of knowledge has accumulated on the characteristics of individuals’ saccadic pursuit during visual attention tasks (Williams and Ford, 2007), little evidence exists to support the *trainability of* visual search (Mann et al., 2007).

A further limitation to the expertise approach is that, for example, the focus has been on a narrow set of conclusions from the original publication on deliberate practice (Ericsson et al., 1993). Therefore, it is not surprising that the debate over the contribution of deliberate practice to expert performance continues in chess (Campitelli and Gobet, 2011; Detterman, 2014), sporting expertise (Williams and Ford, 2007; Baker and Young, 2014) and professional expertise (Ericsson, 2009; Hoffman, 2014). Disagreements over the number of hours accumulated, starting age, and the link to general cognitive abilities continue to dominate the field (e.g., Gobet and Campitelli, 2007; Baker and Young, 2014; Hambrick et al., 2014a). For example, Hambrick et al. (2014b, p. 113) concluded that evidence had accumulated to suggest that, although relevant, deliberate practice, in itself, “does not largely account for individual differences in performance.” One caveat to be considered here is that these authors were concerned with the predictive ability of the theory solely within the context of chess. The acquisition of motor skills in the traditional sporting context is arguably more complex (Voss et al., 2010). For example, even defining deliberate practice among athletes is more challenging than with chess grandmasters (MacIntyre et al., 2013; Healy et al., 2014). Nevertheless, the conclusion by Hambrick et al. (2014b, p. 114) that “the question now is what else matters” suggests that we should consider a broader range of constructs in order to more comprehensively understand expertise.

Before we consider the construct of metacognition, the rationale for studying athletes and sport performers must be made readily apparent. Numerous authors have highlighted the role in which sport can provide a natural laboratory for the study of constructs within psychology and expertise (Ericsson and Smith, 1991; Moran, 1996; MacIntyre et al., 2013). According to Ericsson (2009, p. 18) “performance can be publically observed and even objectively measured in open competition and public performances.” Similarly, it has been noted that the high performance sport environment is dynamic. For example, typically performers have to execute complex skills under conditions of extreme stress where their limits are being constantly challenged (Baker and Young, 2014). Among the topics that have only recently received scrutiny are the role of attention and the allocation of effort in deliberate practice (Baker and Young, 2014). One explanation for this is that researchers concentrated on the variables that were most measurable, including the quantification of hours in practice (Helsen et al., 1998). A challenge for researchers has been reconciling the automaticity and procedural knowledge, central to expert sport performance, with the notion that declarative knowledge and metacognitive abilities may also play a role in the acquisition of expertise (Stanley and Krakauer, 2013; Toner, 2014). To explain, while procedural knowledge is inherently linked to optimum sport performance, declarative knowledge may have both a debilitative (Beilock and Carr, 2001) and facilitative role (Carson and Collins, 2011; MacIntyre et al., 2013; Brick et al., 2014). For example, it is probable that Carl Lewis knew his precise stride count to enable him to hit the board and take off into the sandpit at the 1984 Olympic Games. Thus, expertise in sports goes beyond mere procedural knowledge and arguably metacognitive processes are present at all stages of the target skill and may work in parallel. We thus propose an integrative model of expertise in sports, one that explores action and cognition in sport, a topic that has arguably only recently returned to the forefront of psychology.

In Rosenbaum (2005) suggested that researchers in psychology have historically turned relatively late to cognition and action. While this point is debatable, given the recent emergence of research on exceptional performance states (e.g., Choking; Beilock and Carr, 2001), the paucity of research on action in prior decades may be worthy of review. Understanding movement had long been the preserve of the fields of motor control, biomechanics, and neurophysiology perhaps due to the complexity of the cognition-action nexus and the lack of clear methodological approaches within psychology (Rosenbaum, 2005).“Thinking and action seem to lie at opposite ends of the behavioral spectrum” (Moran, 2012b, p. 1). The disembodied approach of information processing theorists in the 1970s led scientists to conduct research on thinking independently from the study of sensorimotor processes and mechanisms (Laakso, 2011). It was not until the advent of the motor cognition paradigm (Jeannerod, 1994) that “action” became subject to intensive scrutiny by researchers in psychology (MacIntyre et al., 2013). Jeannerod proposed that action, rather than movement *per se*, was vital to understand, as evidence for the role of cognition in movement planning was accumulating. The interest in understanding action from different perspectives was increasing rapidly (see Guillot and Collet, 2010). According to Moran (2009) importance of inter-disciplinary collaboration between researchers in cognitive sport psychology, cognitive psychology, and cognitive neuroscience has been brought to the fore by this new paradigm. Similarly, social cognition has developed as a field of study which has added considerably to our understanding of action (Gallese et al., 2004; Frith, 2012). And recently, cognitive researchers have embraced the study of the domain of sport in their quest to understand how the mind works (MacIntyre et al., 2013). Consequently, we propose that metacognition, a construct that is central to motor cognition, social cognition and action, can augment our current explanations and understanding of the preparation and execution of motor skills within the sport context and elucidate our conceptions of expertise.

## WHAT IS METACOGNITION?

Metacognition, or “knowledge or cognition about cognitive phenomena” (Flavell, 1979, p. 906) is curiously under-explored in the domain of expertise among sports performers (Moran, 1996; MacIntyre and Moran, 2010). Elite athletes are not just experts in movement execution but conceivably they are also experts in planning, metacognition, and reflection. Metacognition has been defined as an individual’s insight into and control over their own mental processes (Flavell, 1979). In the decades since Flavell’s (1979) pioneering article, the term metacognition has been operationalized as the scientific study of the mind’s ability to monitor and control itself or, in other words, the study of our ability to know about our knowing (Van Overschelde, 2008, p. 47). It is a different kind of cognition as explained by Nelson (1999, p. 625): “If one aspect of cognition is monitoring or controlling another aspect of cognition, then the former aspect is metacognitive in relation to the latter aspect. Flavell and subsequent investigators have suggested a tripartite model of metacognition, with knowledge, control and monitoring components (Flavell, 1979, 1987; Tarricone, 2011; Halpern, 2014). Recently, Tarricone (2011) indicated that the main interaction between metacognition and self-regulation is to control, monitor, and regulate strategies to meet task demands and goals. Previously, the study of metacognition has targeted intellectual skills and a substantial corpus of research exists on metacognition in educational settings (Hacker et al., 2009). However, Augustyn and Rosenbaum (2005, p. 911) recently challenged the status quo in metacognition research and stated that “if intellectual and perceptual-motor skills rely on similar mechanisms, one would expect metacognition to apply to the guidance of perceptual-motor skills, just as it does to the guidance of intellectual skills.” The approach among cognitive neuroscientists, focused on visual perceptual tasks to measure metacognition (Palmer et al., 2013; Weil et al., 2013), is similarly narrow, perhaps due to methodological issues. Metacognition and action, on the other hand, offers new possibilities in illuminating our understanding of expertise and action.

## EXPERTISE AND METACOGNITION

Expertise is tightly coupled with metacognition in both training (e.g., knowledge of when a skill has been acquired) and competitive settings (e.g., self-regulation under stress). We propose that metacognitive processes are inherently related to expertise in sports and we have summarized recent findings in the sport literature that reflect the prominence of metacognitive explanations (see **Table 1**). Early investigations were focused on judgments of learning (Simon and Bjork, 2001) and more recently more specificity in the research questions has led to the development of specific models (MacIntyre and Moran, 2010). In the coming sections, we postulate that people use different sources of information, including metacognitive inferences. Firstly, we contend that expertise in any given area facilitates metacognitive inference and secondly, that expertise itself may consist of metacognitive inference, among a range of other non-metacognitive processes including working memory and motivation. Given that expertise is explained by differences in knowledge, many processes involving the use of that knowledge are more or less automatic or procedularized, and consequently they do may not place onerous demands on working memory (Beilock and Carr, 2001). This creates the opportunity for metacognitive reasoning to optimize the assessment of situations and to structure one’s goal pursuit. Furthermore, experts have quite good ideas about standards and deviations from such standards, whether this refers to one’s own behavior or to the behavior of others. Deviations from sophisticated mental models (e.g., the ideal long jump) are thus more likely to become salient to experts than to non-experts, also providing opportunities for reflective thoughts and interventions. The use of both action simulations (e.g., mental practice) and pre-performance routines by elite performers can be conceptualized as domain-general strategies which rely upon metacognitive processes. Evidence to support these contentions will be presented in the forthcoming sections.

**Table 1:** Recent research in sport that has highlighted the role of metacognition.

Topic	Authors	Method	Emerging literature on metacognition
Meta-imagery	Moran (2002)	Conceptual	Posed the question of whether meta-imagery abilities would distinguish elite from non-elite performers.
Attentional strategies	Nietfield (2003)	Survey	Indicated that the runners relied mostly on monitoring and information management strategy (i.e., strategy thoughts during running) regulatory cognitions.
Meta-imagery	Weinberg et al. (2003)	Survey	Athletes were surveyed, not just on their use of imagery, but on their perceived effectiveness of imagery for distinct functions.
Meta-imagery	MacIntyre and Moran (2007a)	Qualitative	Relative to non-athletes (Denis and Carfantan, 1985), the participants demonstrated an intricate understanding of imagery processes including the use of imagery of realistic behaviors as opposed to ideal performance imagery.
Meta-imagery	MacIntyre and Moran (2007b)	Qualitative	Evidence from elite performers suggested that they possessed sophisticated meta-imagery control skills – being able, for instance, to restructure negative imagery so that it facilitates future performance.
Psychological skills training	Foster and Weigand (2008)	Conceptual	Psychological skills in sport (e.g., imagery, goal setting) can be applied more efficiently, particularly in developmental contexts, by applying metacognitive models to understand the role of self-monitoring and self-regulation in the application of the above strategies.
Meta-imagery	MacIntyre and Moran (2010)	Conceptual	A model of meta-imagery was proposed with a specific emphasis on expertise effects.
Attention and choking behavior	DeCaro et al. (2011)	Experimental	Skill failure was linked to the extent to which skill execution depends on explicit attentional control. Increased metacognitive awareness may cause performers to evaluate their performance diverting attention away from skill execution.
Meta-imagery	Pearson et al. (2011)	Experimental	Findings support the role of meta-cognitive knowledge of imagery ability and relate it to our ability to judge individual episodes of imagery.
Attention and ironic processes	Toner et al. (2013)	Experimental	Over-compensatory behavior was more prevalent amongst low-skilled than high-skilled golfers and they concluded that future research explore metacognition.
Attentional strategies	Brick et al. (2014)	Conceptual	The authors in developing a tentative framework for attentional focus in endurance activity, highlighted the potential benefits of applying a metacognitive approach in future studies.

### THE ROLE OF METACOGNITIVE INFERENCES

The Coliseum, Los Angeles, XXIII Olympic Games, August 8th 1984. A strong wind was swirling around the stadium in the afternoon as Carl Lewis’s was preparing for his long jump (50 stunning Olympic moments No. 44: Carl Lewis’s four golds in 1984, 2012). ABC network commentators were referring to Carl Lewis’ adjustments: “He has to block that out and has to only think about heading down the runway and getting off as long a jump as possible.” The other reporter then stated: “He has got a bit of a headwind. I think that’s what he was waiting for… to decide what he is going to do with his step with regard to this wind.” Carl Lewis ran down the runway and leapt into the history books with a jump that at that stage was 50 cm greater than his rivals. Nevertheless, the commentators stated that it “looked like a very restrained effort to me, the last four or five strides… he really looked like he was sort of in that same stride that he was running in the last 50 m of the 200 this morning [200 m heats]” (Corry, 1984).

After fouling his next jump, Lewis decided not to take his four other allotted jumps and many of the crowd responded by booing despite the margin of victory ultimately being 30 cm (Corry, 1984). Many of the spectators plausibly wanted him to break Bob Beamon’s longstanding world record. However, Lewis’ rationale was that he had other goals to achieve (e.g., to equal Jesse Owens four track and field gold medals in 1936) and he still had his additional rounds to run in the 200 and 100 m relay. Given that Carl Lewis won four gold medals, just as Jessie Owens had, we can conclude that his strategies were successful. Both, the execution of the final jump, in addition to his decision to rest, indicate metacognitive inferences in addition to his athletic expertise.

When individuals perform skills in the sporting context the social situation provides many pieces of information. For example, when Carl Lewis is about to perform a long jump, he may remember his coach’s words about his holding back on his speed both to hit the board accurately and to preserve energy. As he looks down the runway and notices that it is somewhat slippery, and is unsure whether his left foot will begin to ache again, just as it did 30 years ago he is metacognitively engaged. There may be even much more metacognitive activity occurring than the aforementioned examples may suggest. The cognitive system is challenged with the amount of information that is accessible at a given time and the implications that these pieces of information may have for the execution of skill need to be considered. For optimum adjustments, people need to be attuned to thoughts and feelings about the self and the requirements of the social situation. Yet, given that most social situations provide a tremendous amount of information, people need to be able to focus, that is, they need to be selective. So, when it comes to particular tasks such as a long jump, people need to select “what is relevant” and ignore “what is not relevant” for the task at hand. How does this work?

#### A model of metacognition

Bless et al. (2009) proposed a model of metacognition that focuses on different information types. Specifically, cognitively accessible declarative knowledge (i.e., knowledge about something) and feelings (e.g., how people feel about an action) and memories (e.g., whether they can remember a coach’s instruction) serve as information about the situation and the target in question. This has particular resonance when people are uncertain about the judgmental target or when affective states are not in line with implications of the situation, then metacognitive processes are more likely to come into play (e.g., Martin et al., 1993; Bless et al., 2009). Metacognitive inferences are governed by rules or theories that decide whether the accessible information should be used and how it should be used in the moment.

When an expert judges performance, this may access a representation about the target behavior (e.g., a motor image). For example, imagine Carl Lewis watching his 1984 long jumps at the Olympic Games. A vast amount of information about long jumps, such as Bob Beamon’s Leap of the Century at the Olympic Games in Mexico City in 1968, and his own jump might be cognitively accessible at the time. Which pieces of information are relevant? People apply filtering rules, considering whether the information is relevant and representative for the target behavior (e.g., Martin, 1986; Bless et al., 2009). Information might just as easily become accessible in a conversation and thus comprise declarative information. Declarative knowledge is defined as “knowing that” (e.g., taping factual information) and contrasts with procedural knowledge, or skill memory, which is explained as “knowing how” (Sternberg and Sternberg, 2012). For example, Carl Lewis might have a chat with Bob Beamon and Mike Powell about his 1984 long jump performance. If information becomes accessible as part of conversations, the person also needs to ask him or herself whether the information is appropriate to be used for a judgment or decision. Here conversational rules as outlined seem to guide decisions on relevance and informativeness of communicated information (e.g., Schwarz, 1994; Igou and Bless, 2003, 2005, 2007; Bless et al., 2009). Furthermore, metacognitions may relate to past or future affective states. These are thoughts that include many different sources of information (e.g., Wilson and Gilbert, 2003; Igou, 2004, 2008) and assessments of the past or forecasts of future affective states are likely to influence one’s behavior (Kahneman and Snell, 1992). For example, a long jumper may know that he has felt good being in competitions and thus decides to take part in one that is coming up in 2 weeks time.

Another important type of information and likely source of metacognitive processes are people’s feelings at the time of judgments or behavior. For example, a long jumper’s mood, emotion, or the ease with which information comes to mind, may influence the execution of the long jump. Importantly, these feelings have informational value (e.g., Schwarz, 2002) for the judgment or behavior at hand. Usually, affective feelings are distinguished from cognitive feelings (Bless et al., 2009). Moods and emotions are considered affective feelings, and they can inform us about the situation and the target (e.g., Schwarz, 1990). For example, according to Schwarz and Clore (1983), when asked to judge a target (e.g., life in general), people ask themselves “How do I feel about it?” which leads to positive evaluations when people are in a positive mood, and to negative evaluation when they are in a negative mood (e.g., Forgas, 2001).

The feeling of knowing (e.g., Koriat, 1993), ease of retrieval (e.g., Schwarz et al., 1991), familiarity (e.g., Jacoby et al., 1989), and processing fluency (e.g., Reber et al., 1998) are examples of cognitive feelings (e.g., Bless et al., 2009; Huntsinger and Clore, 2011). In a nutshell, these are all experiences that accompany cognitive processes and interact with these processes by serving as information about judgmental targets. For example, according to the ease of retrieval heuristic (Schwarz et al., 1991), if it is easy to think of having performed long jumps, then one would be likely to evaluate one’s jumping capacity more favorably, than if it was difficult to retrieve such examples.

Memories have direct effects on how cognitive representations are formed. However, congruent with Strack and Bless (1994) and Bless et al. (2009), we argue that people also use theories about the functioning of memory as an indicator as to whether accessible information is valid and relevant for a judgment at hand. For example, Carl Lewis may not remember that the coach warned him to run within himself for the long jump run-up. Possibly, Carl would reason that he would remember that because it would have been very untypical for his coach to hold this opinion.

Our conceptualization of metacognition is in line with a broad definition of the construct, namely that metacognition is cognition about both thoughts and feelings. However, the narrower definition of metacognition as control process (e.g., Shea et al., 2014) is also just as valid. The latter refers to processes in which attention and cognitive control are essential in structuring peoples’ thoughts and actions. As Shea et al. (2014) describe in detail, these types of thoughts are associated with cognitive effort and limited capacity (cognitive system 2) rather than automatic, effortless processes (cognitive system 1; Stanovich, 2011).

Generally, monitoring and excluding accessible items of information requires cognitive resources. As a result, reduction in cognitive capacity increases the likelihood that relatively irrelevant information is used for the judgment task at hand (Bless et al., 2009). To be clear, we do not think that metacognitions always need awareness and controlled thought processes; however, we believe that metacognitive inferences are especially influential when people need to engage in this type of reflective behavior in order make a judgment or decision. This is especially the case when situations are ambiguous and complex, when more or less automatic processes fail, or when accessible information is conflicting, contradictory, or perceived as inappropriate for the task at hand.

### PST AND METACOGNITION: THE CASE FOR DOMAIN-GENERAL SKILLS

Interestingly, the ability of Carl Lewis to combine excellence in both track (e.g., 100, 200 m sprints and relay) and field events (e.g., long jump) supports the domain-specificity of expertise. To explain, long jump performance is determined largely by the athlete’s ability to attain a fast horizontal speed at the end of the approach runway, thus the physiological task demands were compatible (Bridgett and Linthorne, 2006). However, it is also likely that what are termed “domain-general skills” including psychological skills and metacognition may have played a role in his nine Olympic gold medal winning performances.

As noted earlier (see **Table 1**), Foster and Weigand (2008) highlighted that some theoretical inadequacies in sport psychology could be reconciled by considering other conceptual frameworks, including meta-cognitive approaches (Flavell, 1979). For example, by augmenting our understanding of psychological skills in sport with the construct of metacognition, we could more clearly understand the role of self-monitoring and self-regulation in the application of the aforementioned strategies.

Moran (1996) suggested that PST in sport is essentially an exercise in meta-cognitive instruction. Thus, in order to help athletes become independent thinkers, we need to know what they know and believe about how their own minds work. In this regard, meta-cognitive control processes are especially valuable because they allow people to change their behavior strategically in accordance with task demands. Eccles and Feltovich (2008) proposed that accelerated learning and enhanced performance, and ultimately expertise, may be the result of a combination of psychological support skills (e.g., self-talk, goal-setting, relaxation, and mental practice) and metacognitive abilities. They are domain-general in that they “can be applied to a variety of novel tasks and domains” (Eccles and Feltovich, 2008, p. 43). Meta-cognitive skills in this case are higher order skills that regulate learning and performance, including the coordination of the use of psychological support skills (i.e., PST).

Within sport psychology, psychological skills have been shown to differentiate successful Olympians from their less successful counterparts (Orlick and Partington, 1988; Gould et al., 2002; Fletcher and Sarkar, 2012). The coordinating role of metacognition may be a key factor in the efficient use of psychological skills. Furthermore, emotional regulation is trainable and sustainable by the application of PST and this has applications beyond the realm of sport (Eccles et al., 2011). Two other aspects of psychological skills that are indeed trainable are now discussed, meta-imagery and routines.

#### Is meta-imagery linked to expertise?

One dimension of metacognition that has been illuminated by recent research activity is “meta-imagery,” a performer “beliefs about the nature and regulation of their own imagery skills” (Moran, 2002, p. 415). In 2002, little was known about this topic relative to the knowledge base on other aspects of imagery, such as motor imagery. Over the preceding years research in the expertise literature emerged to suggest that meta-imagery is another factor that differentiates novices from experts (Moran et al., 2012). Researchers had explored the topic by asking athletes to indicate why, where, how, what, and when they use mental imagery processes (e.g., Munroe et al., 2000; MacIntyre and Moran, 2007a,b). Athletes’ responses from both interviews and surveys demonstrated a comprehensive knowledge of the multi-modal potential of imagery, showed they employed imagery in creative ways for contingency planning (see Moran, 2009) and were also aware of robust imagery effects (e.g., mental practice). Interestingly, a meta-analysis conducted in 1994, indicated a possible constraint on the efficacy of mental practice for novice learners (i.e., experts improved more). Driskell et al. (1994) suggested that novices may not have an appropriate approximation of the motor skill or that their imagery abilities may be insufficient to generate and manipulate the requisite visuo-spatial motor configuration. An alternative possibility is that experts may simply possess greater meta-cognitive knowledge of how to employ imagery effectively for skill improvement as compared to novices (MacIntyre et al., 2013). In fact, a model of meta-imagery was developed to account for the above findings and this also generates possibilities of developing a test of meta-imagery (MacIntyre and Moran, 2010).

Furthermore, contemporary evidence from cognitive psychology supports the role of meta-cognitive knowledge of imagery ability and relates it to our ability to judge individual episodes of imagery (Pearson et al., 2011). The voluntary nature of imagery and the role of conscious awareness during imagery tasks make it amenable to introspection, ironically the method that was central to the demise of the scientific study of imagery, a century ago (Roeckelein, 2004).

#### Is winning just a matter of routine?

Pre-performance routines are integral to performance excellence in many self-paced sporting skills, from sprint running to penalty taking in field games (Singer, 2000, 2002; Jackson and Baker, 2001). Defined by Moran (1996, p. 177) as “a sequence of task-relevant thoughts and actions which an athlete engages in systematically prior to his or her performance of a specific sports skill.” The widespread use of routines in sport demonstrates that attention is central to cognitive sport psychology because the ability to exert mental effort effectively is vital for optimal athletic performance (Moran, 2009). One function of routines is to regulate arousal prior to skill execution and evidence for their role in buffering stress or choking has also emerged (Mesagno and Mullane-Grant, 2010). While routines have been explored across a range of sports, perhaps, the sport of golf has received the most attention from researchers (e.g., McCann et al., 2001; Cotterill et al., 2010). Interestingly, three time major winner golfer Padraig Harrington is quoted as saying “my key isn’t working at the moment so we have to figure out a way… I have gone a bit stale focusing on the target.” What is instructive about this statement is that the golfer appears to realize that his routine is no longer functioning appropriately. Routines need to be revised regularly to avoid the routine itself becoming too automatic (Moran, 1996). From a metacognitive perspective, this may be accounted for by metacognitive monitoring. Thus metacognition may be fundamental to the refinement of pre-performance routines as well as their acquisition. A recent review noted that “at a fundamental level it is still not clear what function routines fulfill, what they should consist of or the most effective way to teach them” (Cotterill, 2010, p. 132). The potential for metacogniton research to shed light on the development and refinement of routines as well as their theoretical and conceptual basis is readily apparent.

### NEW AVENUES FOR FUTURE RESEARCH

In the preceding paragraphs, arguments for the potential for the construct of metacognition to clarify our understanding of expertise have been made. Now we wish to specify research topics augmented by appropriate methodologies and possible tasks (see **Table 2**).

**Table 2:** Proposed research topics, methods, and objectives of future studies to study expertise and metacognition in sport settings.

Research topic	Methods	Objectives
Measurement	Psychometric and experimental approach	To engage in conceptual analysis of the construct of metacognition (and related constructs). To investigate, using dual-task methods, the distinct cognitive processes underlying metacognition (e.g., interference with aspects of working memory). To create psychometrically valid measures of metacognition and action processes (e.g., meta-imagery).
Motor cognition	Action simulation and converging methods	To generate specific hypotheses to empirically test if metacognition is a domain-general skill across different motor simulation processes. To use paradigms from motor cognition (e.g., mental travel studies) to evaluate metacognitive monitoring ability.
Anxiety	Experimental and field study approach	To investigate stereotype threat and the interaction with metacognitive processes in both well-learned and novel skills. To examine how current affect and anticipated affective responses to performance influence action in sports via meta-cognitive thoughts. To examine how metacognitive training can influence skilled performance and athletes’ susceptibility to overcompensation of attention.
Neuroscience	Neural imaging	To investigate the neural substrates of the existing models of cognitive control that relate to metacognition processes. To specify the neural architecture underlying metacognitive abilities. To elucidate whether metacognition is linked to a global mechanism or if distributed neural substrates underlie different components of metacognition.
Developmental	Mixed-methods	To assess the role of cognitive development in the acquisition of meta-cognitive skills. To explore the potential of interventions to enhance metacognitive abilities among those who experience deficits in, for example, their judgments of learning. To understand the interaction between metacognitive abilities and motor skill acquisition across the lifespan (e.g., how the elderly can cope using metacognitive skills to supplement diminishing working memory).

#### Measurement

One of the key challenges in the operationalization of any construct is the development of appropriate measurement tools. At present, there is a paucity of questionnaires to assess metacognitive abilities. One such measure is the 52-item *Metacognitive Awareness Inventory* (Schraw and Dennison, 1994) which employs a two factor model (knowledge of cognition; regulation of cognition). Currently, there is a need to develop and validate revised psychometric instruments to assess, for example, meta-imagery beliefs and knowledge. Further questionnaires for meta-attention or social metacognition (for team sports) could also be piloted, refined and analyzed using factor analysis. Parallel with the objective would be the refinement of the construct of metacognition as it relates to both expertises. Tarricone (2011) had conducted a comprehensive “taxonomy of metacognition” which is primarily focused upon on the wide-scale research in the educational research domain. A similar task would be beneficial for metacognition within the context of expertise and the models discussed in this review (e.g., Bless et al., 2009). The approach of Fleming and Lau (2014, p. 1) who distinguish between “metacognitive *bias* (a difference in subjective confidence despite basic task performance remaining constant), metacognitive *sensitivity* (how good one is at distinguishing between one’s own correct and incorrect judgments) and metacognitive *efficiency* (a subject’s level of metacognitive sensitivity given a certain level of task performance)” raises interesting questions for the study of metacognition and conscious awareness. Their approach, focusing on perceptual expertise, includes elements which have a direct relevance to expert performance. For instance, they suggest that “metacognitive confidence” can be interpreted as a probability judgment directed toward one’s own decisions-the probability of a previous judgment being correct. This is synonymous with expertise (i.e., ability to predict actions). It is our view that the exploration of the construct of metacognition and how they interface with action related processes (e.g., motor imagery), we can contribute to the conceptual development of the construct of metacognition. The divergent approaches to date necessitate a degree of conceptual analysis, a process that is all too rare in psychology (Machado and Silva, 2007).

#### Motor cognition

Recent conceptualizations of imagery, action observation and motor execution, view these processes as overlapping, differing by degree rather than kind (Jeannerod, 1994, 2006; Vogt et al., 2013). The preceding section on meta-imagery is illustrative of the progress that can be made in our understanding of expertise, metacognition and imagery, alike. Given the overlap between for example, imagery (e.g., visualizing a long jump-the run-up, take-off, and landing phases) and action observation (e.g., viewing Bob Beamons’ world record long jump), a question arises as to whether the same metacognitive processes underlie these related processes. This issue is further complicated by evidence from several sources which suggests motor imagery is grounded in physical experience, for example, the specific training either simulated or executed (Olson and Nyberg, 2011; Debarnot et al., 2014). The question remains as to whether the respective metacognitive processes are domain-general or domain-specific? As a result, deeper conceptual analysis is required to comprehensively describe and explain the range of metacognitive processes that pertain to cognitive simulation strategies. This new dimension to metacognition research offers a range of experimental possibilities that can enable a greater understanding of metacognition with regard to action preparation, simulation, and execution.

#### Anxiety

Research on “choking” in sport has illuminated our understanding of anxiety across both cognitive skills (e.g., Lyons and Beilock, 2012) and motor skill contexts (e.g., Beilock and Carr, 2001; Beilock and Gonso, 2008; DeCaro et al., 2011; Toner and Moran, 2011; Toner et al., 2013). The “explicit monitoring hypothesis” suggests that attending to a well learned skill may lead to failure in the precise execution of the skill under pressure. Metacognitive abilities obviously have a role in regulation emotion, based on the aforementioned model by Bless et al. (2009). Furthermore, the role of “stereotype threat” which occurs when “knowledge of a negative stereotype about a social group leads to a les-than-optimal performance by members of that group” should be investigated from a metacognitive perspective (Beilock and McConnell, 2004). Previous investigations supported the contention that stereotype threat prompts attention to the executed action and thus can disrupt performance (Beilock et al., 2003). However, this effect can be alleviated by the inclusion of a secondary task. Findings across two studies conducted by Beilock et al. (2003) were inconsistent and the impact of stereotype threat may be more telling across a tournament than a putting skill as it may interfere with the metacognitive processes that help modulate attention and regulate emotion. Another avenue is to systematically examine how current affect and anticipated affective responses to performance influence action in sports via meta-cognitive thoughts. This is based on the recent literature on affect regulation (e.g., Baumeister et al., 2007; Loewenstein, 2011) Moreover, it would be interesting to investigate how training in metacognition can influence skilled performance and athletes’ susceptibility to overcompensation of attention and affect regulation.

#### Neuroscience

The rise of neuroscience in recent decades has been based largely upon advances in methodologies that facilitate the study of internal mental experiences, such as metacognition, in a robust and scientific way. Cognitive neuroscience, in particular, has had a dramatic effect on our understanding of individual domains of cognition from vision to memory (Beran et al., 2012), in chess (Bilalic et al., 2010, 2012; Bartlett et al., 2013) and more recently in sport (Debarnot et al., 2014). As we have seen throughout the current paper sport and athletic skills offer a dynamic and fascinating arena to study and explore cognitive processes, metacognitive processes, and experiences.

Regarding expertise, we saw that in order to engage in effective training episodes for long periods of time, athletes must be highly self-disciplined and self-regulated (Crews et al., 2001). This notion of self-regulation, defined as a set of cognitive, behavioral, and motivational processes that interact to influence performance (Kitsantas and Kavussanu, 2011) has been the go-to approach for examining expertise differences in performance domains. This approach has been concerned with self-regulatory processes (imagery, attentional control, for example) and researchers typically attempted to make confidence judgments about the efficacy of some aspect of their cognition. Neuroscience has enabled researchers to move beyond the study of processes and focus on metacognitive judgments instead (a case in point being; the feeling of inaccessibility otherwise known as the tip of the tongue phenomena). This temporary failure of retrieval for a memory highlights a problem with a particular cognitive process but not a problem with one’s metacognitive judgment. What tip of the tongue research has shown us is that different underlying processes are responsible for the cognition and metacognition that monitors it (Schwartz and Diaz, 2014). Metacognitive experiences arise from cognitive processes and correspond to particular behaviors. The cognitive processes that produced the behavior are not the same as the processes that gave rise to the metacognition. For example, an object is recognized as having been seen before (cognitive process) accompanied by an experience of confidence, and the person then says that they know the answer (behavior). Essentially, there are a set of cognitive processes driving the recall of information but another set of processes driving our awareness of it. “Thus understanding any metacognitive judgment must involve understanding the cognition it measures and the multiple processes that contribute to the judgment” (Schwartz and Diaz, 2014, p. 9). Attempts at componential analysis of metacognition are in their infancy (Fleming and Lau, 2014; Garrison, 2014), but they appear to be fruitful with regard to understanding its impact upon visual perceptual tasks. Nevertheless, the investigation of the neural basis of metacognition (Baird et al., 2013) is not without its limitations. It has been noted that the application of neurophysiological measurement techniques impose restrictions on the ecological validity of studies which are not readily overcome (Mann et al., 2013).

#### Developmental

Currently, a gap exists in our knowledge of how performers acquire pre-performance routines (Singer, 2000, 2002; Cotterill, 2010). Unfortunately, researchers have neglected to explore how these strategies are developed over time with one recent notable exception, a study with gymnastics athletes (Faggiani et al., 2012). The role of cognitive development in the acquisition of meta-cognitive skills may be a limiting factor for applied sport psychology interventions (Foster and Weigand, 2008). Thus the gap in the knowledge base may be due to the complex interaction of domain-general and domain-specific cognitive skills. Given that our approach has centered on the role of metacognitive abilities and processes, we propose that a developmental approach to understanding pre-performance routines could be augmented by exploring metacognitive skill development from a longitudinal perspective. The potential of specific interventions to enhance metacognitive ability could be explored for those who are impaired in their metacognitive development or for those who suffer plateaus in their skill development. For example, recent research has demonstrated the ability of a 2-week meditation program in enhancing metacognitive ability in a perceptual task (Baird et al., 2014).

## CONCLUSION

Do metacognitive processes enhance performance? Are they helpful in the acquisition of expertise? The general answer to these questions is in the affirmative. Metacognitive processes are part of the inventory of human thought (Nelson and Narens, 1994; Sternberg, 2001; Perfect and Schwartz, 2002). As such, they serve as a resource to structure thought and regulate behavior. Will metacognitive processes always lead to better outcomes? No, certainly not. As for all areas of information processing, people can err. However, understanding the role of metacognition, the breadth and flexibility of processes involved and how they are associated with expertise, allows for more precise predictions of behavior.

We argue that more research and empirical scrutiny of the construct of metacognition can help to develop principles that govern the relation between internal cognitive processes and subjective experience. These principles could be very effective for expertise research looking to differentiate a “real” expert from a “skilled” performer, currently a major challenge in expertise research (e.g., MacIntyre et al., 2013; Bourne et al., 2014).

In the sporting domain, training athletes to initiate, develop and engage in metacognition can equip them with the proper strategies, beliefs, and self-understanding to excel in sports. Currently, there is no unified view as to what athletic training entails and coaches, despite a burgeoning literature (Healy et al., 2014) have, for example, focused on a relatively narrow set of conclusions from the deliberate practice literature (Baker and Young, 2014). Metacognition offers the potential to expand our understanding of expertise and individual domains of cognition through a rigorous examination of the mechanisms underlying self-initiated monitoring and control of ones own performance. Consequently, our understanding of expertise can be illuminated by studying metacognition in the sporting, domain, specifically, by using a strength-based approach with expert samples (MacIntyre et al., 2013). Sport offers researchers a fertile natural laboratory where expertise is easily quantifiable through the quest to be faster, higher and stronger. In conclusion, we have demonstrated that the construct of metacognition has the potential to be a springboard for research into sporting expertise.

## Conflict of Interest Statement

The authors declare that the research was conducted in the absence of any commercial or financial relationships that could be construed as a potential conflict of interest.
